# Identification of a Five-Gene Signature and Establishment of a Prognostic Nomogram to Predict Progression-Free Interval of Papillary Thyroid Carcinoma

**DOI:** 10.3389/fendo.2019.00790

**Published:** 2019-11-15

**Authors:** Mengwei Wu, Hongwei Yuan, Xiaobin Li, Quan Liao, Ziwen Liu

**Affiliations:** Department of General Surgery, Peking Union Medical College Hospital, Chinese Academy of Medical Sciences & Peking Union Medical College, Beijing, China

**Keywords:** TCGA, GEO, papillary thyroid carcinoma, progression-free interval, nomogram

## Abstract

**Background:** The incidence of papillary thyroid carcinoma (PTC) is high and increasing worldwide. Although prognosis is relatively good, it is important to select the minority of patients with poorer prognosis to avoid side effects associated with unnecessary over-treatment in low-risk patients; this requires accurate prognostic predictions.

**Materials and Methods:** Six PTC expression datasets were obtained from the gene expression omnibus (GEO) database. Level 3 mRNA expression and clinicopathological data were obtained from The Cancer Genome Atlas Thyroid Cancer (TCGA–THCA) database. Through integrated analysis of these datasets, highly reliable differentially-expressed genes (DEGs) between tumor and normal tissue were identified and lasso Cox regression was applied to identify DEGs related to the progression-free interval (PFI) and to establish a prognostic gene signature. The performance of a five-gene signature was evaluated based on a Kaplan–Meier curve, receiver operating characteristic (ROC), and Harrell's concordance index (C-index). Multivariate Cox regression analysis was used to identify factors associated with PTC prognosis. Finally, a prognostic nomogram was established based on the TCGA-THCA dataset.

**Results:** A novel five-gene signature was established to predict the PTC PFI, which included *PLP2, LYVE1, FABP4, TGFBR3*, and *FXYD6*, and the ROC curve and C-index showed good performance in both training and validation datasets. This could classify patients into high- and low-risk groups with distinct PFIs and differentiate PTC tumors from normal tissue. Univariate Cox regression revealed that this signature was an independent prognostic factor for PTC. The established nomogram, incorporating the prognostic gene signature and clinical parameters, was able to predict the PFI with high efficiency. The gene signature-based nomogram was superior to the American Thyroid Association (ATA) risk stratification to predict PTC PFI.

**Conclusions:** Our study identified a five-gene signature and established a prognostic nomogram, which were reliable in predicting the PFI of PTC; this could be beneficial for individualized treatment and medical decision making.

## Introduction

The incidence of thyroid cancer is highest among endocrine tumors, and has been increasing over the past 20 years; it is now the eighth-most commonly diagnosed cancer in the world (1). Differentiated thyroid cancer accounts for the most prevalent thyroid cancer and among this type, thyroid papillary cancer (PTC) and follicular thyroid cancer have relatively good prognoses with long term survival rates higher than 90% (2). Further, PTC accounts for ~85% of thyroid cancers and by 2030, it will rank fourth among the most common malignant tumors in the United States (3, 4). A variety of risk factors such as radiation are associated with the onset of differentiated thyroid cancer. Moreover, exposure to ionizing radiation during childhood or adolescence can lead to the development of PTC. Iodine deficiency and Hashimoto's thyroiditis were also reported to be associated risk factors for thyroid cancer (5).

The *BRAF* V600E mutation is the most common mutation in PTC. *RAS* mutations are also common, especially in the follicular variant of PTC, which is relatively indolent. *TERT* promoter mutations are reported to be predictive of a worse prognosis for PTC. Although the incidence of thyroid cancer continues to rise, its mortality rate remains low. However, the mechanisms underlying PTC recurrence remain unknown.

Secondary surgery for PTC recurrence results in surgical trauma and a higher risk of recurrent laryngeal nerve injury for patients. Further, 77% of PTC recurrence occurs within 5 years post-surgery (6). Lobectomy or total thyroidectomy is the main treatment for this disease and regular postoperative neck ultrasound examination and the detection of TSH and thyroglobulin levels are the major means to monitor recurrence in patients with postoperative PTC. In addition, patients at a high risk of recurrence and with aggressive tissue subtypes might require radioactive iodine (131I) remnant ablation. Postoperative thyroid cancer patients also undergo routine TSH inhibition therapy to inhibit tumor recurrence and improve prognosis. However, long-term subclinical hyperthyroidism caused by TSH inhibition might lead to a variety of potential side effects such as osteoporosis, atrial fibrillation, cardiac insufficiency, and increased risk of fracture and heart disease in elderly patients (7). Therefore, the challenge for PTC therapy lies in the balance between side effects due to treatment and benefits to patients. Accordingly, the accurate assessment of postoperative PTC prognosis is critical to ensure that low-risk patients are not over-treated, but that high-risk and advanced patients receive necessary and more aggressive therapeutics. The American Thyroid Association (ATA) currently recommends using TNM staging to predict mortality and have also proposed a system to estimate the risk of recurrence (8).

With advances in gene chips and high-throughput sequencing, an increasing number of studies has shown that gene signatures based on mRNA expression levels has great potential to predict PTC prognosis. Choi et al. established a 12-gene predictive model (including *BCC8, CHI3L1, CLCNKA, FAM155B, GABRG1, LUM, MRO, MT1G, MT1H, SELV, SLC4A4*, and *TMEM92*) that might accurately predict nodal metastasis in PTC using data from The Cancer Genome Atlas Thyroid Cancer (TCGA THCA) dataset (9). Moreover, using the TCGA dataset, Lin et al. proposed a seven-gene prognostic signature (including *AGTR1, CTGF, FAM3B, IL11, IL17C, PTH2R*, and *SPAG11A*) based on immune-related genes that might predict the prognosis of PTC (10). Thus, further exploration of public databases such as gene expression omnibus (GEO) and TCGA could reveal additional genes associated with PTC prognosis to establish a reliable prognostic prediction model. Such models combined with clinical pathological parameters might ultimately represent powerful tools to predict PTC prognosis and guide individualized postoperative treatment.

In this study, we integrated six PTC datasets from the GEO database and the TCGA-THCA dataset to identify reliable differentially-expressed genes (DEGs) in PTCs. Further, univariate Cox survival analysis and lasso Cox regression analysis were performed to identify DEGs associated with the progression-free interval (PFI) of PTC, and we proposed a prognostic gene prediction model using gene expression and clinical data from the TCGA-THCA dataset. The molecular mechanisms underlying the gene prediction models were also studied. The potential of this model to differentiate malignant thyroid nodules from normal tissue was also explored. We further applied multivariate Cox survival analysis to identify independent risk factors associated with PTC prognosis. Finally, a nomogram was established combining the gene prediction model with clinical pathological parameters to predict disease outcome. Overall, our new model and nomogram might provide a powerful tool to predict PTC prognosis.

## Materials and Methods

### Gene Expression and Clinical Data

mRNA expression data and related clinical data for PTC were searched and downloaded from GEO (https://www.ncbi.nlm.nih.gov/geo/). The keywords “Thyroid cancer,” “Thyroid carcinoma,” and “PTC” were used for retrieval. Studies based on “*Homo sapiens*” described as “Expression profiling by array” were included for the next round of screening. Studies involving only cases of “follicular thyroid cancer” or “undifferentiated thyroid cancer” were excluded. Studies focusing only on “cell lines” and “xenografts” were also excluded. Finally, six gene expression microarray datasets (GSE5364, GSE29265, GSE33630, GSE35570, GSE38545, GSE60542) were chosen and downloaded for DEG analysis. The selected datasets all met the following criteria: (1)contained human thyroid tissue samples; (2) contained tumor and non-tumor thyroid tissue control samples; (3) contained at least 40 samples. The probes were matched to the gene symbols using the annotation file provided by the manufacturers. The median ranking value was used to calculate the expression value if a single gene symbol was matched by multiple probes. The expression data were normalized based on the Robust Multi-array Average (RMA).

Harmonized RNA sequencing data (HTSeq-counts and HTSeq-FPKM) and associated clinical information for thyroid carcinoma (THCA) were downloaded from TCGA (https://portal.gdc.cancer.gov/, up to June 30, 2019) using *TCGAbiolinks* R package (11), which included 507 cases, 510 tumor samples, and 58 normal tissue samples. After removing five cases without corresponding tumor samples, two cases with the pathological diagnosis of poorly differentiated oncocytic carcinoma or follicular carcinoma, five cases with a history of neoadjuvant therapy, eight samples of metastasis, 495 cases with corresponding tumor tissues and clinical information and 58 normal thyroid tissue samples were ultimately included in the study. Mutation and copy number alteration data were obtained from Cbioportal (http://www.cbioportal.org/) (12). Information regarding *BRAF*-like and *RAS*-like classification proposed by TCGA and was obtain from the official TCGA publication (13).

### Integrated Analysis of Microarray Datasets and Identification of DEGs

For the GEO datasets, DEGs between tumor and normal tissues were identified using the LIMMA package from R software (14). For the TCGA dataset, DEG analysis was applied using *TCGAbiolinks* in R with harmonized RNA sequencing data in the form of HTSeq-counts following the official instruction (11). The cutoff value was set to | Log_2_FC (fold-change) | >1, *p* < 0.05, and false discovery rate (FDR) < 0.05. Integrated analysis of DEGs identified based on six GEO datasets was applied using the robust rank aggregation (RRA) method-based R package “RobustRankAggreg”; *p* < 0.05 was considered statistically significant. The intersection between integrated DEGs from GEO datasets and DEGs of the TCGA-THCA dataset was identified to obtain reliable DEGs indicative of PTC. Gene ontology and KEGG enrichment analyses were applied to explore the potential biological processes, cellular components, molecular functions, and significantly relevant signaling pathways associated with the DEGs using DAVID (https://david.ncifcrf.gov/) (15). *p* < 0.05 was considered statistically significant. The significantly relevant signaling pathways were visualized using Cytoscape v3.7.1 (https://cytoscape.org/).

### Survival Analysis and Establishment of Prognostic Gene Signature

Recurrence and metastasis after initial surgery are the main factors associated with poor outcomes for patients with PTC. Meanwhile, considering the relatively good prognosis and the extremely low risks associated with overall survival, the PFI was chosen as the primary endpoint in this study. All follow-up data were derived from TCGA Pan-Cancer clinical data (16). The TCGA-THCA dataset was used to determine whether the DEGs were associated with the PFI. Normalized gene expression data in the form of Transcripts Per Million (TPM) were transformed based on the base-2 logarithm for further survival analysis. Three cases with follow-up ≤30 days were excluded from the survival analysis. A total of 492 TCGA cases with a follow-up >30 days were randomly and equally divided into a training dataset and a validation dataset. The expression levels of DEGs were then analyzed in the entire TCGA dataset using a univariate Cox proportional hazards regression model. DEGs with a *p* < 0.05 were considered statistically significant and included for further analysis. The training dataset was then used to construct the prognostic gene model. Lasso penalized Cox regression analysis was performed to select prognostic genes associated with the PFI and to construct a prognostic gene signature for patients with PTC based on a linear combination of the regression coefficients derived from the lasso Cox regression model coefficients (β) multiplied by normalized mRNA expression levels. X-Tile software was used to determine the optimal cut-off value of the gene signature (17). Patients were then divided into low- and high-risk groups accordingly. Kaplan–Meier analysis, the area under the (AUC) of the receiver operating characteristic (ROC) curve, and Harrell's concordance index were used to evaluate the performance of the prognostic gene signature. The validation and entire datasets were used for validation. The performance of the gene signature was also compared with the previously defined seven-gene signature proposed by Lin et al. (10). Risk scores for each case were calculated using the same formula and the optimal cut-off value for each dataset was determined using X-Tile software. The performance of the gene signature to differentiate PTC tissues from normal tissues was also tested based on the AUC of the ROC curve.

### Identification of Independent Prognostic Parameters for PTC

To identify independent prognostic parameters for PTC associated with the PFI and to validate the independent prognostic value of the gene signature, univariate and multivariate Cox regression analyses were performed based on the prognostic gene signature and clinical parameters, including *BRAF* V600E mutation status, *RAS* mutation status, *TERT* mutation status, *TERT* expression level, age, histological type, aggressive subtypes, T stage, N stage, M stage, AJCC stage, residual tumor status, extrathyroidal extension, tumor size, multifocality, and the anatomic site of tumors based on the entire TCGA dataset. Parameters with *p* < 0.25 based on univariate analysis were further included in the multivariate Cox regression analysis. *p* < 0.05 was considered statistically significant.

### Building and Validation of a Predictive Nomogram

After testing for collinearity, independent prognostic parameters and relevant clinical parameters were included to construct a prognostic nomogram to predict 1-, 2-, 3-, 4-, and 5-year progression-free survival for PTC patients in the entire TCGA dataset using a stepwise Cox regression model. Kaplan–Meier analysis, AUC of the ROC curve, Harrell's concordance index, and a calibration plot comparing predicted progression-free survival and observed survival were used to evaluate the performance of the prognostic nomogram. Harrell's concordance index was calculated to assess the discrimination of the nomogram based on a bootstrap method with 1,000 replicates. The calibration curve of the nomogram was plotted to compare predicted progression-free survival with observed survival rates. Based on the total points of the nomogram, patients were divided into two or three groups according to the optimal cut-off values determined by X-Tile. Kaplan–Meier analysis was used to plot the survival curves for groups with different risk levels of disease progression.

### Statistical Analysis

Statistical analysis was performed using R software v3.4.3 and GraphPad Prism v8.01 (https://www.graphpad.com). A χ2 test or Fisher's exact test was used to analyze categorical variables. A Student's *t*-test was used to analyze continuous variables for paired samples. One-way ANOVA tests were used to analyze multiple groups of continuous variables. Univariate and multivariate Cox regression analyses were performed for survival analysis. Hazard ratios (HRs) and 95% confidence intervals (CIs) were calculated to identify DEGs associated with progression-free survival. *P* < 0.05 was considered statistically significant unless otherwise indicated.

## Results

### Identification of DEGs

**Figure 1:**
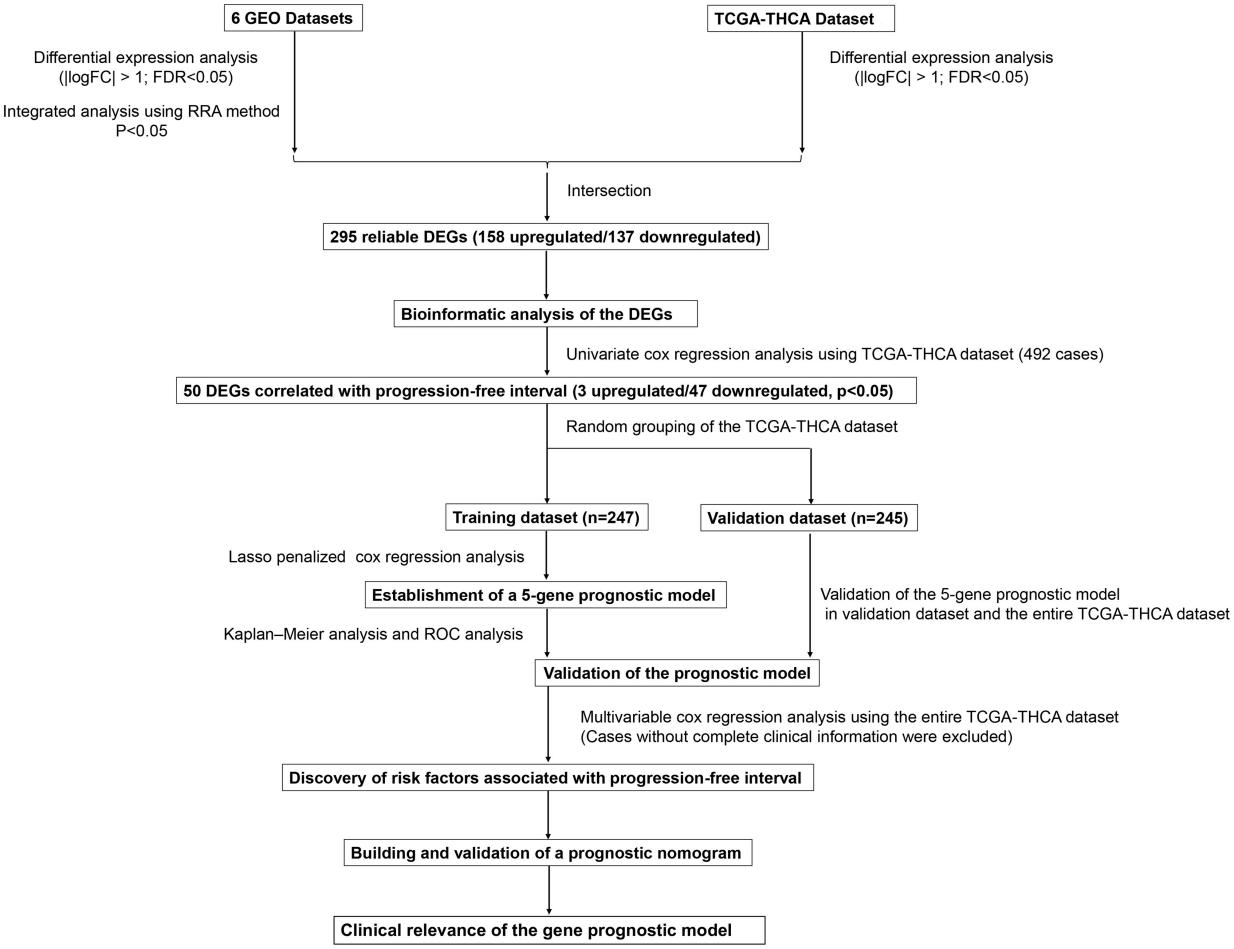
Flowchart presenting the process of establishing the five-gene signature and prognostic nomogram for papillary thyroid carcinoma (PTC).

**Figure 2:**
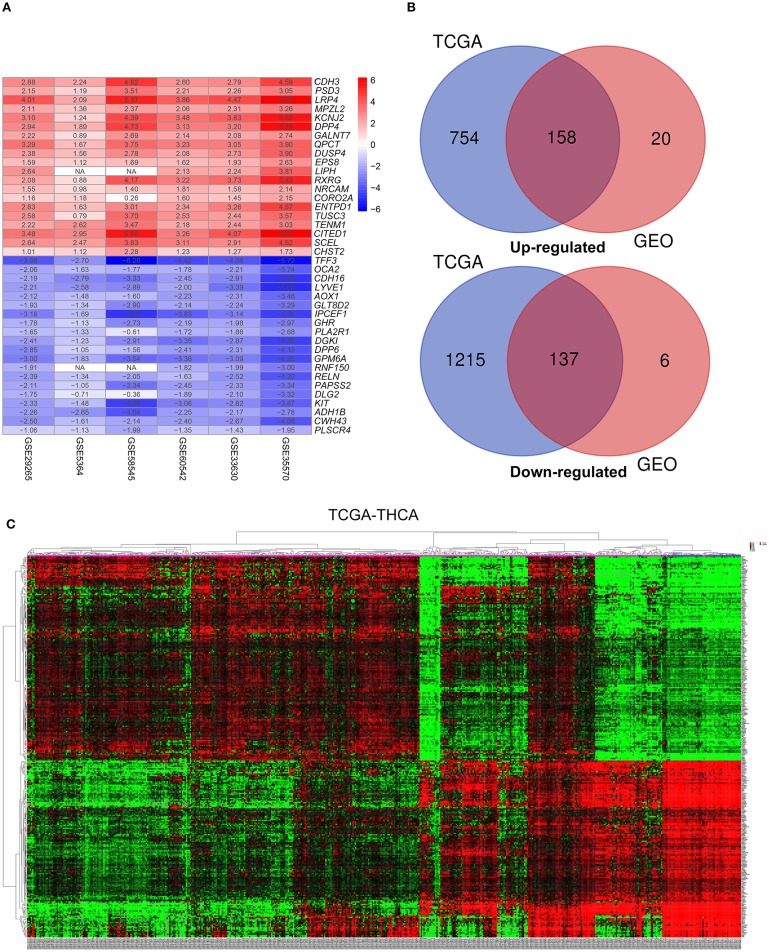
Identification of differentially-expressed genes (DEGs) between tumor tissues and normal tissues in papillary thyroid carcinoma (PTC). **(A)** The heatmap presenting the top 20 upregulated and downregulated DEGs in PTC after integrated analysis of the six GEO datasets using the robust rank aggregation (RRA) method. **(B)** From this, 295 reliable DEGs including 158 upregulated and 137 downregulated genes were identified based on the intersection between GEO and TCGA results. The upregulated DEGs are shown in red while the downregulated DEGs are shown in blue. The value of Log2FC was presented in each column. **(C)** A representative heatmap of TCGA-THCA dataset revealed that the 295 reliable DEGs have significantly different expression patterns between tumor and non-tumor tissues, which can distinguish these tissue types.

This study was carried out based on the flowchart shown in Figure 1. The detailed information regarding the six GEO datasets included in this study is shown in Table 1. In GSE35570, GSE33630, GSE5364, GSE60542, GSE58545, and GSE29265 datasets, a total of 1554, 807, 222, 731, 753, and 558 DEGs, respectively, were identified between tumor and normal thyroid tissues (Supplementary Figure 1). A total of 321 DEGs including 178 upregulated and 143 downregulated genes were identified after the integrated analysis of the GEO datasets using the RRA method. The top 20 upregulated and downregulated DEGs identified by integrated analysis are shown in Figure 2A. In the TCGA-THCA dataset, a total of 2,264 DEGs including 912 upregulated and 1352 downregulated genes were identified (Supplementary Figure 1). Finally, a total of 295 reliable DEGs including 158 upregulated and 137 downregulated genes were identified based on the intersection between GEO and TCGA results (Figure 2B, Supplementary Table 1). Hierarchical cluster analysis showed that the 295 differential genes had significantly different expression patterns between tumor and non-tumor tissues, which could distinguish these tissue types (Figure 2C, Supplementary Figure 2). Functional enrichment analysis of DEGs were showed in Supplementary Figure 3, Supplementary Table 2.

**Table 1:** Details of the GEO datasets included in this study.

**Datasets**	**References**	**Platform**	**Sample size (Tumor/Control)**	**Application**
GSE35570	(18)	[HG-U133_Plus_2] Affymetrix Human Genome U133 Plus 2.0 Array	116 (65/51)	Identification of DEGs
GSE33630	(19)	[HG-U133_Plus_2] Affymetrix Human Genome U133 Plus 2.0 Array	94 (49/45)	Identification of DEGs
GSE60542	(20)	[HG-U133_Plus_2] Affymetrix Human Genome U133 Plus 2.0 Array	63 (33/30)	Identification of DEGs
GSE5364	(21)	[HG-U133A] Affymetrix Human Genome U133A Array	51 (35/16)	Identification of DEGs
GSE58545	(22)	[HG-U133A] Affymetrix Human Genome U133A Array	45 (27/18)	Identification of DEGs
GSE29265	Contributed by Dom et al. (19)	[HG-U133_Plus_2] Affymetrix Human Genome U133 Plus 2.0 Array	40 (20/20)	Identification of DEGs

### Identification of PFI-Related DEGs and Establishment of a Five-Gene Prognostic Signature

**Figure 3:**
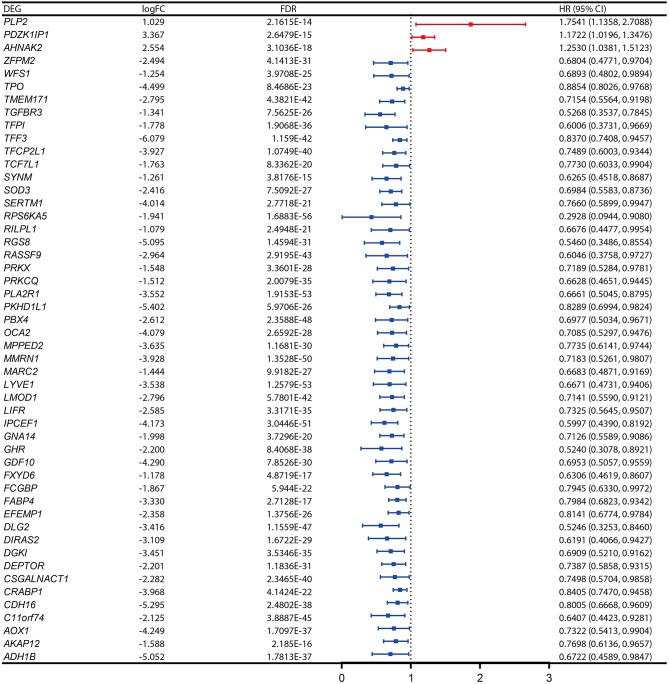
Expression profiles and the forest plot of hazard ratio (HR) showing the prognostic values of the 50 differentially-expressed genes (DEGs) associated with progression-free interval of papillary thyroid carcinoma (PTC).

**Figure 4:**
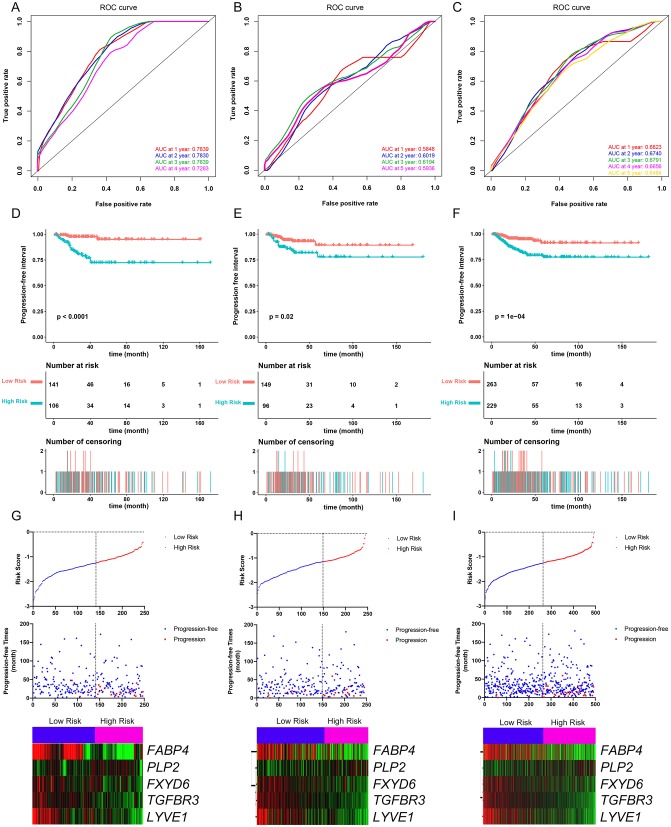
Evaluation of the performance of the five-gene prognostic model in the training dataset and confirmation based on the validation dataset and the entire TCGA-THCA dataset. **(A)** The time-dependent receiver operating characteristic (ROC) for 1-, 2-, 3-, and 4-year progression-free interval (PFI) predictions for the five-gene signature in the training dataset. **(B)** The time-dependent ROC for 1-, 2-, 3-, and 5-year PFl predictions for the five-gene signature in the validation dataset. **(C)** The time-dependent ROC for 1-, 2-, 3-, and 4-year PFI predictions for the five-gene signature in the entire TCGA-THCA dataset. **(D)** The Kaplan–Meier survival curves of the five-gene signature. Patients from the training dataset were stratified into two groups according to the optimal cutoff values for the risk scores calculated by X-Tile software. **(E)** The Kaplan-Meier survival curves of the five-gene signature. Patients from the validation dataset were stratified into two groups according to the optimal cutoff values for the risk scores calculated by X-Tile software. **(F)** The Kaplan–Meier survival curves of the five-gene signature. Patients from the entire TCGA-THCA dataset were stratified into two groups according to the optimal cutoff values for the risk scores calculated by X-Tile software. **(G)** Relationship among the risk score (upper), survival status of patients in different groups (middle), and the expression profiles of the five prognostic genes (bottom) in the training dataset. **(H)** Relationship among the risk score (upper), survival status of patients in different groups (middle), and the expression profiles of the five prognostic genes (bottom) in the validation dataset. **(I)** Relationship among the risk score (upper), survival status of patients in different groups (middle), and the expression profiles of the five prognostic genes (bottom) in the entire TCGA-THCA dataset.

The PFI was chosen as the primary endpoint in this study. A total of 492 TCGA cases with a follow-up period >30 days were included in survival analysis. The included cases were randomly and equally divided into a training dataset and a validation dataset. The baseline characteristics of the patients are shown in Table 2. The entire TCGA dataset was utilized to discover DEGs associated with the PFI of PTC using a univariate Cox regression model. A total of 50 DEGs were identified as associated with the PFI (Figure 3). A prognostic gene signature composed of five genes was then developed based on the training dataset using the lasso Cox penalized regression model, including proteolipid protein 2 (*PLP2*), transforming growth factor beta receptor type 3 (*TGFBR3*), lymphatic vessel endothelial hyaluronic acid receptor 1 (*LYVE1*), FXYD domain-containing ion transport regulator 6 (*FXYD6*), and fatty acid-binding protein, adipocyte (*FABP4*) (Supplementary Figure 4). Among these DEGs, *PLP2* (upregulated) with a HR > 1 was considered an oncogene, whereas *TGFBR3, LYVE1, FXYD6*, and *FABP4* (all downregulated) with HRs < 1 were considered tumor suppressor genes. The risk score was equal to [(0.06066) × normalized expression value of *PLP2*] – [(0.35719) × normalized expression value of *TGFBR3*] – [(0.19667) × normalized expression value of *LYVE1*] – [(0.10089) × normalized expression value of *FXYD6*] – [(0.01634) × normalized expression value of *FABP4*]. A risk score for each case was then calculated according to the formula. X-Tile software was used to determine the optimal cut-off of the risk score. Patients in the training dataset were then divided into high- and low-risk groups accordingly (cut-off value = −1.24). The Kaplan–Meier survival curve revealed significantly worse prognosis in the high-risk group (*p* < 0.0001) (Figure 4D). Next, the prognostic value of the five-gene prognostic signature was assessed based on the time-dependent ROC and C-index. The AUCs of the 1-, 2-, 3-, and 4-year risk scores for PFI prediction were 0.783, 0.783, 0.764, and 0.728, respectively (Figure 4A). The C-index of the risk score was 0.734 (95% CI, 0.653–0.815). Further, the expression of the five genes changed gradually along with the increase in the risk score (Figure 4G). In general, the results indicated good performance for the five-gene signature in predicting the PFI of PTC.

**Table 2:** Baseline characteristics of patients in the TCGA-THCA dataset.

**Clinical features**	**Training dataset**	**Validation dataset**	**Entire TCGA dataset**	***P*-value**
*N*	247	245	492	
Follow-up time (day)	1240.09 ± 989.21	1213.29 ± 1003.87	1226.74 ± 995.61	0.956
Risk score	−1.35 ± 0.45	−1.31 ± 0.43	−1.33 ± 0.44	0.512
Age	46.50 ± 15.70	48.03 ± 16.00	47.26 ± 15.85	0.565
PFI				0.993
Progression-free	222 (89.88%)	221 (90.20%)	443 (90.04%)	
Progression	25 (10.12%)	24 (9.80%)	49 (9.96%)	
*BRAF* V600E				0.896
Wildtype	96 (38.87%)	104 (42.45%)	200 (40.65%)	
Mutant	142 (57.49%)	135 (55.10%)	277 (56.30%)	
NA	9 (3.64%)	6 (2.45%)	15 (3.05%)	
*RAS* mutation				0.962
Wildtype	209 (84.62%)	209 (85.31%)	418 (84.96%)	
Mutant	29 (11.74%)	30 (12.24%)	59 (11.99%)	
NA	9 (3.64%)	6 (2.45%)	15 (3.05%)	
*TERT* mutation				0.906
Wildtype	229 (92.71%)	222 (90.61%)	451 (91.67%)	
Mutant	16 (6.48%)	19 (7.76%)	35 (7.11%)	
NA	2 (0.81%)	4 (1.63%)	6 (1.22%)	
*TERT* expression	0.05 ± 0.18	0.07 ± 0.28	0.06 ± 0.24	0.660
Sex				0.857
Male	69 (27.94%)	63 (25.71%)	132 (26.83%)	
Female	178 (72.06%)	182 (74.29%)	360 (73.17%)	
Histological type				0.895
Thyroid Papillary Carcinoma—Classical/usual	181 (73.28%)	169 (68.98%)	350 (71.14%)	
Thyroid Papillary Carcinoma—Follicular (≥ 99% follicular patterned)	49 (19.84%)	52 (21.22%)	101 (20.53%)	
Thyroid Papillary Carcinoma—Tall Cell (≥ 50% tall cell features)	15 (6.07%)	19 (7.76%)	34 (6.91%)	
Others	2 (0.81%)	5 (2.04%)	7 (1.42%)	
T				0.967
T1	75 (30.36%)	67 (27.35%)	142 (28.86%)	
T2	76 (30.77%)	85 (34.69%)	161 (32.72%)	
T3	84 (34.01%)	83 (33.88%)	167 (33.94%)	
T4	12 (4.86%)	9 (3.67%)	21 (4.27%)	
NA	0 (0.00%)	1 (0.41%)	1 (0.20%)	
N				0.874
N0	104 (42.11%)	120 (48.98%)	224 (45.53%)	
N1	29 (11.74%)	29 (11.84%)	58 (11.79%)	
N1a	44 (17.81%)	44 (17.96%)	88 (17.89%)	
N1b	41 (16.60%)	31 (12.65%)	72 (14.63%)	
NA	29 (11.74%)	21 (8.57%)	50 (10.16%)	
M				0.737
M0&Mx	241 (97.57%)	241 (98.37%)	482 (97.97%)	
M1	6 (2.43%)	3 (1.22%)	9 (1.83%)	
NA	0 (0.00%)	1 (0.41%)	1 (0.20%)	
AJCC stage				0.915
Stage I	133 (53.85%)	146 (59.59%)	279 (56.71%)	
Stage II	24 (9.72%)	26 (10.61%)	50 (10.16%)	
Stage III	57 (23.08%)	51 (20.82%)	108 (21.95%)	
Stage IV	32 (12.96%)	21 (8.57%)	53 (10.77%)	
NA	1 (0.40%)	1 (0.41%)	2 (0.41%)	
Residual tumor				0.855
R0	184 (74.49%)	191 (77.96%)	375 (76.22%)	
Rx	13 (5.26%)	17 (6.94%)	30 (6.10%)	
R1	31 (12.55%)	20 (8.16%)	51 (10.37%)	
R2	3 (1.21%)	1 (0.41%)	4 (0.81%)	
NA	16 (6.48%)	16 (6.53%)	32 (6.50%)	
Extrathyroidal extension				0.959
None	165 (66.80%)	160 (65.31%)	325 (66.06%)	
Minimal (T3)	62 (25.10%)	70 (28.57%)	132 (26.83%)	
Moderate or Advanced (T4)	9 (3.64%)	8 (3.27%)	17 (3.46%)	
NA	11 (4.45%)	7 (2.86%)	18 (3.66%)	
Multifocality				0.980
Unifocal	130 (52.63%)	128 (52.24%)	258 (52.44%)	
Multifocal	113 (45.75%)	111 (45.31%)	224 (45.53%)	
NA	4 (1.62%)	6 (2.45%)	10 (2.03%)	
Anatomic site				0.666
Unilateral	197 (79.76%)	183 (74.69%)	380 (77.24%)	
Isthmus	13 (5.26%)	9 (3.67%)	22 (4.47%)	
Bilateral	35 (14.17%)	50 (20.41%)	85 (17.28%)	
NA	2 (0.81%)	3 (1.22%)	5 (1.02%)	

### Validation of the Performance of the Five-Gene Prognostic Signature in Predicting PFI

The validation and entire TCGA datasets were then used to validate the performance of the five-gene prognostic signature in predicting PFI. A risk score for each case was calculated using the same formula. The optimal cut-off value was also determined for each dataset using X-Tile software. Patients in each dataset were then divided into high- and low-risk groups accordingly. Kaplan–Meier survival curves revealed that PFIs were significantly distinct between high- and low-risk groups in both datasets (Figures 4E,F). Specifically, patients in the high-risk groups had notably poorer prognosis than those in the low-risk groups. The prognostic predictive power of the five-gene signature was then assessed based on the time-dependent ROC and C-index. In the validation dataset, the AUCs for 1-, 2-, 3-, and 5-year PFI prediction based on the gene signature were 0.584, 0.602, 0.619, and 0.593, respectively, and the C-index of the gene signature was 0.603 (95% confidence interval(CI): 0.484, 0.722) (Figure 4B). In the entire TCGA dataset, the AUCs for 1-, 2-, 3-, 4-, and 5-year PFI prediction based on the gene signature were 0.662, 0.674, 0.679, 0.666, and 0.648, respectively, and the C-index of the gene signature was 0.667 (95% CI: 0.593, 0.741) (Figure 4C). The performance of the five-gene signature was also compared with the previously defined seven-gene signature proposed by Lin et al. The five-gene signature had a comparable prognostic value (C-index, 0.667 vs. 0.632) (Supplementary Figures 5A–E). The distributions of the risk scores and gene expression data are shown in Figures 4H,I. Collectively, validation results indicated that the five-gene signature had good performance in predicting the PFI of PTC patients.

**Figure 5:**
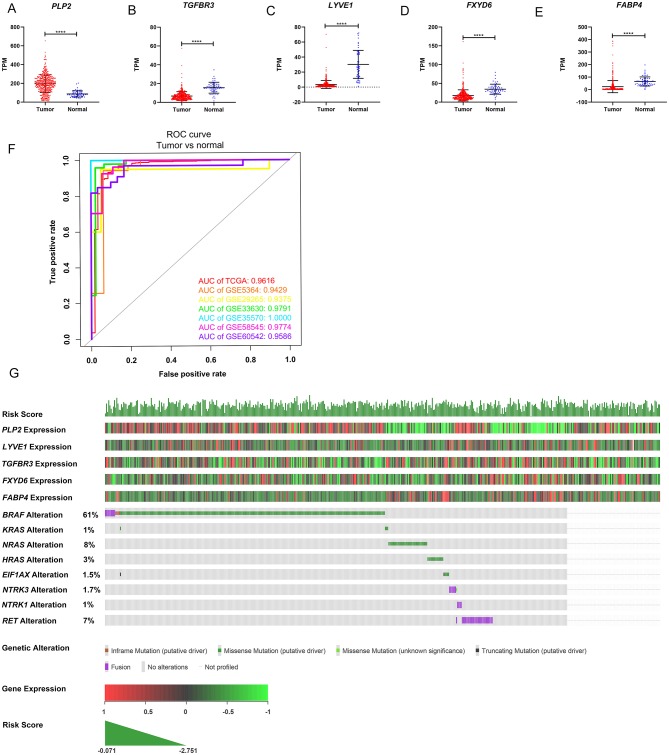
Expression level of the five genes in papillary thyroid carcinoma (PTC) and the mutation landscape of PTC. **(A–E)** Show the mRNA expression levels of the five genes in the PTC tumor tissues and normal tissues of the TCGA-THCA dataset. **(F)** Receiver operating characteristic (ROC) curve showing the performance of the five-gene signature in differentiating normal thyroid tissue from PTC tissue in the GEO and TCGA datasets. **(G)** The relationship among the 5-gene signature, transcriptome profiles and mutational profiles (*BRAF, RAS, EIF1AX, NTRK3*-fusion, *NTRK1*-fusion, *RET*-fusion and *BRAF*-fusion) of PTC. Data were obtained from the cBioPortal for Cancer Genomics (https://www.cbioportal.org). ^*^p < 0.05, ^**^p < 0.01, ^***^p < 0.001, ^****^p < 0.0001.

The gene expression levels of the five genes were explored in the TCGA dataset (Figures 5A–E). The performance of the five-gene signature in differentiating normal thyroid tissue from PTC tissue was evaluated in the GEO and TCGA datasets based on the ROC analysis. The AUCs of the gene signature based on the TCGA, GSE5364, GSE29265, GSE33630, GSE35570, GSE58545, and GSE60542 datasets were 0.962, 0.943, 0.938, 0.979, 1.000, 0.977, and 0.959, respectively, indicating its potential diagnostic value (Figure 5F). The relationship between the 5-gene signature, transcriptome profiles and mutational profiles (*BRAF, RAS, EIF1AX, NTRK3*-fusion, *NTRK1*-fusion, *RET*-fusion and *BRAF*-fusion) of PTC were also analyzed and presented in Figure 5G.

### Evaluation of Prognostic Factors Associated With the PFI in PTC

Patients (376/492) from the entire TCGA-THCA dataset with complete clinical information including *BRAF* V600E and *RAS* mutation status, *TERT* mutation status, *TERT* expression level, sex, age, histological type, TNM stage, residual tumor, extrathyroidal extension, tumor size, multifocality, and the anatomic site of tumors were included to identify prognostic factors (Table 3). Details of exclusion from the further analysis of each case are listed in Supplementary Table 3. Univariate and multivariate Cox regression analyses were applied to identify prognostic factors associated with the PFI in PTC. Univariate analysis showed that risk score, age, *TERT* mutation status, *TERT* expression level, T stage, N stage, M stage, AJCC stage, and the largest dimension of the neoplasm were significantly associated with the PFI (*p* < 0.05) (Table 4). Parameters associated with *P*-values < 0.25 based on univariate analysis were further included in the multivariate Cox regression analysis. Multivariate analysis revealed that risk score (*p* = 0.0077) and *RAS* mutation status (*p* = 0.0129) were independent risk factors (Table 5).

**Table 3:** Baseline characteristics of patients included for the evaluation of prognostic factors and establishment of nomogram.

**Clinical features**	**Mean + *SD***
Follow-up time (day)	1258.24 ± 968.52
Risk Score	−1.30 ± 0.45
Age	47.19 ± 15.59
	N (%)
**PFI**	
Progression-free	338 (89.89%)
Progression	38 (10.11%)
***BRAF*** **V600E**	
Wildtype	143 (38.03%)
Mutant	233 (61.97%)
***RAS*** **mutation**	
Wildtype	333 (88.56%)
Mutant	43 (11.44%)
***TERT*** **mutation**	
Wildtype	344 (91.49%)
Mutant	32 (8.51%)
*TERT* expression	0.06 ± 0.16
**Sex**	
Male	101 (26.86%)
Female	275 (73.14%)
**Histological type**	
Thyroid Papillary Carcinoma—Classical/usual	270 (71.81%)
Thyroid Papillary Carcinoma—Follicular (≥ 99% follicular patterned)	71 (18.88%)
Thyroid Papillary Carcinoma—Tall Cell (≥ 50% tall cell features)	28 (7.45%)
Others	7 (1.86%)
**T**	
T1	109 (28.99%)
T2	121 (32.18%)
T3	129 (34.31%)
T4	17 (4.52%)
**N**	
N0	187 (49.73%)
N1	54 (14.36%)
N1a	74 (19.68%)
N1b	61 (16.22%)
**M**	
M0&Mx	370 (98.40%)
M1	6 (1.60%)
**AJCC stage**	
Stage I	210 (55.85%)
Stage II	38 (10.11%)
Stage III	85 (22.61%)
Stage IV	43 (11.44%)
**Residual tumor**	
R0	313 (83.24%)
Rx	20 (5.32%)
R1	40 (10.64%)
R2	3 (0.80%)
**Extrathyroidal extension**	
None	255 (67.82%)
Minimal (T3)	107 (28.46%)
Moderate or Advanced (T4)	14 (3.72%)
Neoplasm largest dimension(cm)	2.81 ± 1.58
**Multifocality**	
Unifocal	204 (54.26%)
Multifocal	172 (45.74%)
**Anatomic site**	
Unilateral	294 (78.19%)
Isthmus	18 (4.79%)
Bilateral	64 (17.02%)

*Patients from the entire TCGA-THCA dataset without complete clinical information were excluded*.

**Table 4:** Unadjusted univariate analysis.

**Exposure**	**Statistics**	**PFI**
Risk score	−1.30 ± 0.45	4.15 (1.88, 9.16) 0.0004
***BRAF*** **V600E**		
Wildtype	143 (38.03%)	1.0
Mutant	233 (61.97%)	0.99 (0.51, 1.91) 0.9679
***RAS*** **mutation**		
Wildtype	333 (88.56%)	1.0
Mutant	43 (11.44%)	1.91 (0.84, 4.34) 0.1232
***TERT*** **mutation**		
Wildtype	344 (91.49%)	1.0
Mutant	32 (8.51%)	3.25 (1.49, 7.11) 0.0031
*TERT* expression	0.06 ± 0.16	6.30 (2.25, 17.61) 0.0004
**Sex**		
Male	101 (26.86%)	1.0
Female	275 (73.14%)	0.78 (0.39, 1.55) 0.4830
Age	47.19 ± 15.59	1.02 (1.00, 1.04) 0.0449
**Age**		
≤ 55 years	264 (70.21%)	1.0
>55 years	112 (29.79%)	2.31 (1.22, 4.38) 0.0101
**Histological type**		
Thyroid Papillary Carcinoma—Classical/usual	270 (71.81%)	1.0
Thyroid Papillary Carcinoma—Follicular (≥ 99% follicular patterned)	71 (18.88%)	0.76 (0.29, 1.98) 0.5763
Thyroid Papillary Carcinoma—Tall Cell (≥ 50% tall cell features)	28 (7.45%)	2.16 (0.83, 5.62) 0.1135
Others	7 (1.86%)	0.00 (0.00, Inf) 0.9966
**Aggressive subtype**		
No	347 (92.29%)	1.0
Yes	29 (7.71%)	2.30 (0.90, 5.92) 0.0834
**T**		
T1	109 (28.99%)	1.0
T2	121 (32.18%)	2.59 (0.84, 8.05) 0.0989
T3	129 (34.31%)	3.78 (1.28, 11.17) 0.0162
T4	17 (4.52%)	5.51 (1.38, 22.10) 0.0160
**N stage**		
N0	187 (49.73%)	1.0
N1	189 (50.27%)	2.20 (1.11, 4.36) 0.0238
**N1b**		
No	315 (83.78%)	1.0
Yes	61 (16.22%)	2.07 (0.97, 4.40) 0.0585
**M**		
M0&Mx	370 (98.40%)	1.0
M1	6 (1.60%)	5.36 (1.64, 17.52) 0.0055
**AJCC stage**		
Stage I	210 (55.85%)	1.0
Stage II	38 (10.11%)	1.22 (0.35, 4.28) 0.7574
Stage III	85 (22.61%)	2.72 (1.26, 5.87) 0.0108
Stage IV	43 (11.44%)	4.20 (1.79, 9.86) 0.0010
**Residual tumor**		
R0	313 (83.24%)	1.0
Rx	20 (5.32%)	1.36 (0.32, 5.73) 0.6719
R1	40 (10.64%)	1.74 (0.72, 4.18) 0.2183
R2	3 (0.80%)	3.21 (0.43, 23.74) 0.2541
**Extrathyroidal extension**		
None	255 (67.82%)	1.0
Minimal (T3)	107 (28.46%)	1.62 (0.84, 3.15) 0.1524
Moderate or Advanced (T4)	14 (3.72%)	1.72 (0.40, 7.33) 0.4666
Neoplasm largest dimension	2.81 ± 1.58	1.23 (1.03, 1.46) 0.0209
**Neoplasm largest dimension**		
≤ 2 cm	146 (38.83%)	1.0
>2 cm	230 (61.17%)	3.32 (1.39, 7.96) 0.0070
**Multifocality**		
Unifocal	204 (54.26%)	1.0
Multifocal	172 (45.74%)	0.92 (0.48, 1.76) 0.7958
**Anatomic site**		
Unilateral	294 (78.19%)	1.0
Isthmus	18 (4.79%)	0.47 (0.06, 3.46) 0.4598
Bilateral	64 (17.02%)	1.01 (0.42, 2.43) 0.9798

**Table 5:** Multivariate cox regression analysis.

**Exposure**	**Non-adjusted**	**Adjust I**	**Adjust II**	**Adjust III**
Risk score	4.15 (1.88, 9.16) 0.0004	3.39 (1.59, 7.24) 0.0016	3.39 (1.59, 7.24) 0.0016	3.01 (1.34, 6.76) 0.0077
***BRAF*** **V600E**				
Wildtype	1.0	1.0	1.0	NA
Mutant	0.99 (0.51, 1.91) 0.9679	0.87 (0.45, 1.70) 0.6911	0.76 (0.38, 1.49) 0.4183	NA
***RAS*** **mutation**				
Wildtype	1.0	1.0	1.0	1.0
Mutant	1.91 (0.84, 4.34) 0.1232	2.23 (0.97, 5.13) 0.0585	1.97 (0.84, 4.59) 0.1188	4.22 (1.36, 13.11) 0.0129
***TERT*** **mutation**				
Wildtype	1.0	1.0	1.0	1.0
Mutant	3.25 (1.49, 7.11) 0.0031	2.40 (1.02, 5.63) 0.0442	2.11 (0.87, 5.11) 0.0993	1.09 (0.36,3.26) 0.8782
*TERT* expression	6.30 (2.25, 17.61) 0.0004	5.29 (1.46, 19.10) 0.0111	6.39 (1.70, 23.97) 0.0060	4.57 (0.67,31.19) 0.1210
**Sex**				
Male	1.0	1.0	1.0	NA
Female	0.78 (0.39, 1.55) 0.4830	0.95 (0.47, 1.93) 0.8824	0.96 (0.47, 1.97) 0.9137	NA
Age	1.02 (1.00, 1.04) 0.0449	1.00 (0.97, 1.03) 0.7877	1.00 (0.97, 1.03) 0.8325	0.98 (0.94,1.03) 0.4590
**Age**				
≤ 55 years	1.0	1.0	1.0	1.0
>55 years	2.31 (1.22, 4.38) 0.0101	1.36 (0.62, 2.97) 0.4390	1.37 (0.62, 3.03) 0.4352	1.88 (0.52,6.82) 0.3346
**Histological type**				
Thyroid Papillary Carcinoma—Classical/usual	1.0	1.0	1.0	1.0
Thyroid Papillary Carcinoma—Follicular (≥ 99% follicular patterned)	0.76 (0.29, 1.98) 0.5763	0.83 (0.32, 2.19) 0.7105	0.87 (0.33, 2.28) 0.7746	0.40 (0.10, 1.63) 0.1995
Thyroid Papillary Carcinoma—Tall Cell (≥ 50% tall cell features)	2.16 (0.83, 5.62) 0.1135	1.62 (0.60, 4.38) 0.3456	1.38 (0.50, 3.83) 0.5323	0.16 (0.00, Inf) 1.0000
Others	0.00 (0.00, Inf) 0.9966	0.00 (0.00, Inf) 0.9965	0.00 (0.00, Inf) 0.9970	0.00 (0.00, Inf) 0.9963
**Aggressive subtype**				
No	1.0	1.0	1.0	1.0
Yes	2.30 (0.90, 5.92) 0.0834	1.68 (0.63, 4.50) 0.3040	1.41 (0.52, 3.88) 0.5013	9.44 (0.00, Inf) 0.9999
**T**				
T1	1.0	1.0	1.0	1.0
T2	2.59 (0.84, 8.05) 0.0989	2.52 (0.77, 8.23) 0.1244	2.24 (0.69, 7.26) 0.1801	0.86 (0.17, 4.35) 0.8519
T3	3.78 (1.28, 11.17) 0.0162	2.52 (0.79, 8.11) 0.1199	2.24 (0.71, 7.14) 0.1712	1.55 (0.28, 8.59) 0.6152
T4	5.51 (1.38, 22.10) 0.0160	2.14 (0.44, 10.47) 0.3486	1.74 (0.36, 8.44) 0.4922	0.99 (0.07, 14.88) 0.9949
**N stage**				
N0	1.0	1.0	1.0	1.0
N1	2.20 (1.11, 4.36) 0.0238	1.71 (0.77, 3.79) 0.1859	1.72 (0.78, 3.78) 0.1756	2.00 (0.74, 5.36) 0.1704
**N1b**				
No	1.0	1.0	1.0	1.0
Yes	2.07 (0.97, 4.40) 0.0585	1.30 (0.48, 3.51) 0.6030	1.67 (0.62, 4.50) 0.3072	2.12 (0.69, 6.54) 0.1896
**M**				
M0&Mx	1.0	1.0	1.0	1.0
M1	5.36 (1.64, 17.52) 0.0055	4.20 (1.12, 15.76) 0.0335	4.33 (1.14, 16.49) 0.0315	2.32 (0.25, 21.83) 0.4628
**AJCC stage**				
Stage I	1.0	1.0	1.0	1.0
Stage II	1.22 (0.35, 4.28) 0.7574	1.31 (0.34, 5.13) 0.6959	1.18 (0.30, 4.61) 0.8120	1.20 (0.19, 7.39) 0.8449
Stage III	2.72 (1.26, 5.87) 0.0108	2.98 (1.07, 8.30) 0.0366	2.46 (0.88, 6.85) 0.0847	2.25 (0.62, 8.20) 0.2194
Stage IV	4.20 (1.79, 9.86) 0.0010	4.55 (1.52, 13.63) 0.0068	4.07 (1.31, 12.59) 0.0149	1.56 (0.31, 7.79) 0.5910
**Residual tumor**				
R0	1.0	1.0	1.0	1.0
Rx	1.36 (0.32, 5.73) 0.6719	0.96 (0.22, 4.14) 0.9539	0.92 (0.21, 3.98) 0.9111	0.96 (0.16, 5.87) 0.9650
R1	1.74 (0.72, 4.18) 0.2183	1.19 (0.48, 2.94) 0.7067	1.19 (0.48, 2.95) 0.7042	1.02 (0.37, 2.80) 0.9669
R2	3.21 (0.43, 23.74) 0.2541	5.14 (0.43, 61.82) 0.1970	3.62 (0.30, 44.06) 0.3137	7.06 (0.33, 152.03) 0.2119
**Extrathyroidal extension**				
None	1.0	1.0	1.0	1.0
Minimal (T3)	1.62 (0.84, 3.15) 0.1524	1.11 (0.54, 2.28) 0.7797	1.02 (0.49, 2.12) 0.9525	0.70 (0.22, 2.28) 0.5583
Moderate or Advanced (T4)	1.72 (0.40, 7.33) 0.4666	0.61 (0.12, 2.96) 0.5378	0.62 (0.13, 3.00) 0.5484	1.19 (0.10, 13.84) 0.8887
Neoplasm largest dimension	1.23 (1.03, 1.46) 0.0209	1.12 (0.94, 1.34) 0.2008	1.12 (0.92, 1.35) 0.2577	0.81 (0.56, 1.17) 0.2586
**Neoplasm largest dimension**				
≤ 2cm	1.0	1.0	1.0	1.0
>2cm	3.32 (1.39, 7.96) 0.0070	3.22 (1.33, 7.76) 0.0093	2.90 (1.20, 7.01) 0.0185	3.73 (0.82, 16.98) 0.0880
**Multifocality**				
Unifocal	1.0	1.0	1.0	NA
Multifocal	0.92 (0.48, 1.76) 0.7958	0.80 (0.41, 1.57) 0.5154	0.94 (0.47, 1.86) 0.8554	NA
**Anatomic site**				
Unilateral	1.0	1.0	1.0	NA
Isthmus	0.47 (0.06, 3.46) 0.4598	0.51 (0.07, 3.74) 0.5040	0.49 (0.07, 3.66) 0.4896	NA
Bilateral	1.01 (0.42, 2.43) 0.9798	0.79 (0.32, 1.93) 0.6016	1.00 (0.40, 2.48) 0.9975	NA

*Adjust I model adjust for: Age, Sex, and AJCC Stage. Adjust II model adjust for: Age, Sex, AJCC Stage, and Risk Score. Adjust III model adjust for parameters with P < 0.25 based on univariate analysis*.

### Building and Validation of a Prognostic Nomogram

**Figure 6:**
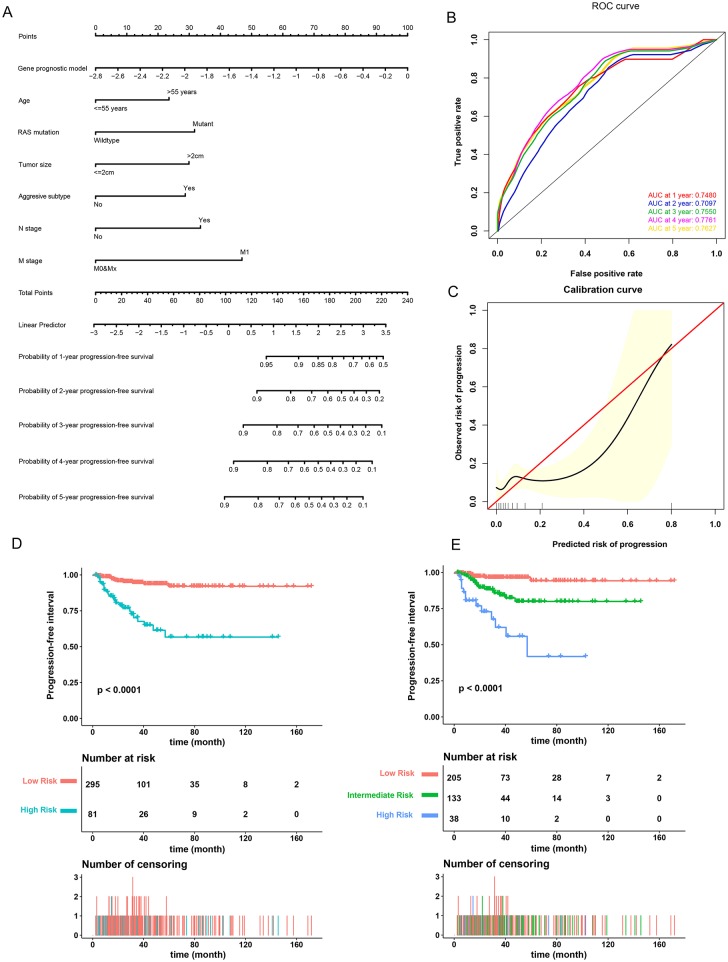
Validation of the nomogram in predicting progression-free interval (PFI) of papillary thyroid carcinoma (PTC) in the TCGA-THCA dataset. **(A)** A prognostic nomogram predicting 1-, 2-, 3-, 4-, and 5-year PFI of PTC. Aggressive subtypes include hobnail, tall cell and columnar. **(B)** Time-dependent ROC showing the 1-, 2-, 3-, 4-, and 5-year PFI predictions of PTC for the nomogram. **(C)** The calibration plot for internal validation of the nomogram. The Y axis represents the actual PFI while the X axis represents the predicted PFI. **(D,E)** The Kaplan–Meier survival curves of the nomogram. Patients from the TCGA-THCA dataset were stratified into two or three groups of different levels of risk according to the optimal cutoff values for the nomogram calculated by X-Tile software.

A prognostic nomogram to predict the 1-, 2-, 3-, 4-, and 5-year PFI of PTC patients was established based on the 376 patients with complete clinical information from the TCGA-THCA dataset using the stepwise Cox regression model (Figure 6A). Risk score, age, *RAS* mutation status, tumor size, aggressive subtype, N stage, and M stage were parameters included in the nomogram. The AUCs for the 1-, 2-, 3-, 4-, and 5-year PFI were 0.7480, 0.7097, 0.7550, 0.7761, and 0.7627, respectively (Figure 6B). The C-index was 0.7600 (95% CI, 0.6759, 0.8440). The patients were then divided into two or three groups associated with different levels of risk based on the cut-off value determined by X-Tile software. Groups with a lower risk score were associated with better prognosis (Figures 6D,E). The calibration curve further revealed that the nomogram had good performance in predicting the PFI of PTC patients (Figure 6C). When the risk of progression was less than 0.15, the nomogram might overestimate the risk but when the risk of progression is greater than 0.15, the nomogram might underestimate the risk.

**Figure 7:**
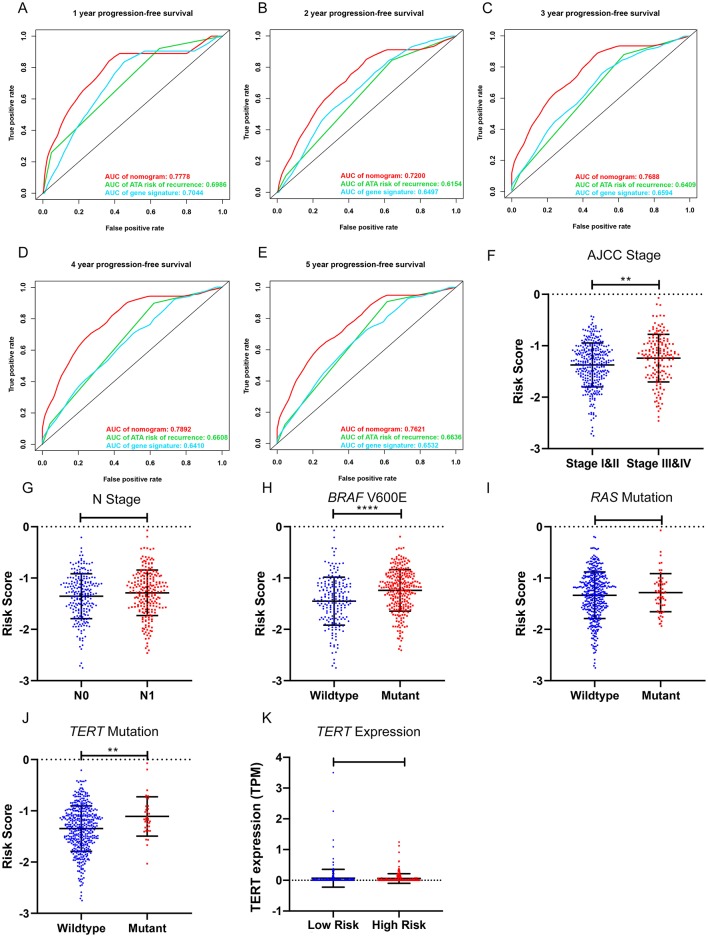
Clinical relevance of the five-gene signature and the prognostic nomogram. **(A–E)** The prognostic performance of the five-gene prognostic model, the gene signature-based nomogram, and the 2009 version of American Thyroid Association (ATA) risk stratification of recurrence. **(F,G)** The distribution of the five-gene risk score based on different AJCC stages and N stages in the TCGA-THCA dataset. **(H–J)** The distribution of the five-gene risk score based on different mutation status of *BRAF* V600E, *RAS* and *TERT* in TCGA-THCA dataset. **(K)** The expression level of *TERT* in high-risk and low-risk group of TCGA-THCA dataset. ^*^p < 0.05, ^**^p < 0.01, ^***^p < 0.001, ^****^p < 0.0001.

To compare the prognostic performance of the gene signature-based nomogram to the currently available risk stratification system, the estimated risk of tumor recurrence based on the 2009 American Thyroid Association guidelines for each case was retrieved from the TCGA integrated genomic data of PTC (16). For this, 346 cases with complete clinical information for the nomogram and sufficient information for ATA risk stratification from the entire TCGA-THCA dataset were included to compare prognostic performances. The AUCs to predict the 1-, 2-, 3-, 4-, and 5-year PFI for the nomogram were 0.7778, 0.7200, 0.7688, 0.7892, and 0.7621 respectively (Figures 7A–E). In contrast, the AUCs for 1-, 2-, 3-, 4-, and 5-year PFI prediction based on ATA risk stratification were 0.6986, 0.6154, 0.6409, 0.6608, and 0.6636, respectively. The C-index of the nomogram was 0.7747 (95% CI, 0.6870–0.8625), whereas the C-index of ATA risk stratification was 0.6377 (95% CI, 0.5601–0.7153), we also compare the prognostic performance of the nomogram with the 2015 version of modified risk stratification proposed by the American Thyroid Association (Noted that modification involving lymph node size is not included) (8). The MACIS score was also used as control. The risk score had the highest C-index (0.7747 vs. 0.6449 and 0.6507) indicating a superior prognostic value (Supplementary Figures 6A–E). The results indicated that the gene signature-based nomogram was superior to ATA risk stratification and MACIS in predicting the PFI of PTC.

The performance of the gene signature-based nomogram was further explored in different subgroups of *BRAF*-like and *RAS*-like PTCs proposed by TCGA (13). In compare with the ATA risk stratification and the previously defined seven-gene signature, the gene signature-based nomogram had the best prognostic performance in all the subgroups (Supplementary Figures 7B–G). The performance of both gene signatures was limited in the *RAS*-like subgroup indicating the potential role of *RAS* mutation as an independent prognostic factor.

### Clinical Relevance of the Gene Signature

The relationships between the gene signature and clinical parameters were then analyzed. In terms of tumor stage, patients with stage III and IV PTC had significantly higher risk scores than patients with stage I and II disease (Figure 7F). The risk scores of patients with lymph node metastasis were higher than those for patients without lymph node metastasis; however, this was not statistically significant (Figure 7G). In terms of mutation status, patients with the *BRAF* V600E mutation had significantly higher risk scores than those without this alteration, whereas risk scores were comparable between patients with and without *RAS* mutations (Figures 7H,I). Patients with *TERT* mutation also had significantly higher risk scores than the wildtype. But the *TERT* expression level was comparable between high-risk and low-risk group (Figures 7J,K). We further explored the difference of the 5-gene signature between *BRAF*-like, *RAS*-like and the Others proposed by TCGA. The risk scores were comparable between *BRAF*-like group and *RAS*-like group. But the *BRAF*-like group has significant higher risk score than the Others (Supplementary Figure 7A).

## Discussion

The incidence of PTC is high and increasing worldwide, resulting in a heavy disease burden on a global scale. Although the prognosis of PTC is relatively good, patients with recurrent PTC still suffer from additional surgical trauma and are at higher risk of surgical complications such as recurrent laryngeal nerve injury (2). In contrast, PTC patients with low recurrence risk suffer from long-term subclinical hyperthyroidism caused by unnecessary postoperative TSH inhibition therapy, which can lead to multiple potential side effects such as osteoporosis, atrial fibrillation, and cardiac insufficiency. Traditional clinicopathological parameters such as TNM staging can predict the mortality associated with PTC, but it is difficult to accurately estimate the risk of recurrence (8). The ATA recurrence risk stratification can predict the risk of recurrence for thyroid cancer, but the accuracy needs to be further improved. Moreover, it does not reflect the biological progression of PTC. The accurate prediction of prognosis for patients with PTC will help to select patients that could benefit from more aggressive treatments including a wider range of surgical treatments, I131 treatment, and a higher degree of TSH inhibition. It also allows patients with a low risk of recurrence to avoid unnecessary I131 treatment and TSH inhibition therapy. Therefore, treatment can be individualized to improve PTC prognosis and improve patient quality of life. Gene signatures can be quantified by standardized detection means, vary with the biological progression of the tumor, and can dynamically reflect prognosis as the patient's condition changes using such approaches. Thus, it might be more accurate and convenient to predict patient prognosis and risk of recurrence. In addition, these prognostic genes could play an important role in the progression of PTC and might represent potential targets to inhibit recurrence and metastasis. Combined with the detection of tumor-associated exosomes and circulating tumor cells (CTCs), the real-time detection of disease recurrence and response to treatment in patients with PTC after tumor resection can be achieved. Because these prognostic genes are closely related to the development of this disease, they are also potential markers for differential diagnosis and the evaluation of biological characteristics of tumors. Active surveillance of thyroid papillary microcarcinoma is currently advocated (23). Predicting the biological characteristics of tumors based on gene signatures through fine needle aspiration could make active surveillance safer. Further, PTC is a highly heterogeneous disease and tumor progression involves a complex network comprising multiple signaling pathways. Therefore, the combination of multiple genes can more accurately reflect the biological characteristics and prognosis of PTC, rather than a single marker. Nomograms are widely used in oncology to evaluate clinical prognosis as they integrate multiple prognostic determinants including molecular biology and clinicopathological parameters to estimate the individual numerical probabilities of clinical events (24). Accordingly, personalized medicine can be achieved. Compared to a conventional staging system, nomograms might predict prognosis more accurately and are easier for patients to understand. Therefore, they could contribute to clinical decision making.

In this study, 321 reliable DEGs in PTC were identified based on the integrated analysis of GEO and TCGA datasets. Survival analysis revealed that 50 DEGs were closely associated with the PFI of PTC. A novel five-gene signature was then established using lasso-Cox regression analysis to predict the PFI of PTC based on a training dataset (TCGA dataset). Among these genes, *PLP2* was upregulated and positively associated poorer survival, whereas *LYVE1, FABP4, TGFBR3*, and *FXYD6* were downregulated and identified as tumor suppressor genes. The five-gene signature was able to classify patients into groups with distinct PFIs and was an independent prognostic factor for PTC. Patients in the high-risk group had a significantly poorer prognosis than patients in the low-risk group. The prognostic performance of the five-gene signature was also confirmed based on the validation dataset and the entire TCGA dataset using AUC and C-index parameters. The five-gene signature also had good performance in differentiating PTC tissues from normal tissues. Moreover, a prognostic nomogram was established based on the five-gene signature and clinical pathological parameters to predict the 1-, 2-, 3-, 4-, and 5-year PFI of PTC.

Among this five-gene signature, two were previously reported to be associated with PTC. *LYVE1* acts as a hyaluronic acid transporter and is involved in the catabolism of lymphatic endothelial cells and transport of substances (25). It is also considered a marker of lymphatic vessels (26). The upregulated expression of *LYVE1* in tumor tissues indicates tumor-associated lymphangiogenesis and was reported to be associated with worse prognosis in breast cancer, renal cancer, and lung cancer (27–29). *LYVE1* might also play a tumor suppressor role. In hepatocellular carcinoma, its expression was demonstrated to decrease progressively from cirrhotic nodules to cancer tissues (30, 31). In prostate cancer, *LYVE1* was found to be downregulated and associated with the relapse of localized prostate cancer (32). Its downregulation was also identified in ovarian cancer and was associated with poorer survival (33). In PTC, current study results suggested that the downregulation of *LYVE1* is associated with worse prognosis. In accordance with our study, *LYVE1* was previously reported to be downregulated in PTC tumors based on microarrays and this result was confirmed by qPCR and IHC (34). However, Gao et al. reported that the expression of steroid receptor coactivator-1 (SRC-1), a potential oncogene, is positively associated with *LYVE1* and associated with lymphatic metastasis in PTC (35). Thus, the role of *LYVE1* in PTC and its relationship with lymph node metastasis remains to be elucidated.

*FABP4* is a lipid transporter in adipocytes that binds long-chain fatty acids and retinoic acid, presenting these molecules to their receptors in the nucleus (36). In accordance with the results of our study, *FABP4* was identified as a tumor suppressor in multiple cancers. In colorectal cancer, *FABP4* was found to be downregulated and its upregulation inhibited the migration, invasion, and proliferation of cancer cells (37). In lung cancer, it was determined that the expression of *FABP4* can be induced by the transcriptional activity of *PPAR*γ, mediating lipolysis and tumor growth suppression (38). Further, in invasive breast cancer, the loss of *FABP4* expression is associated with a higher risk of progression (39). In contrast, *FABP4* plays an oncogenic role in hepatocellular carcinoma, promoting proliferation and migration via downregulation of the HIF1 pathway (40). In ovarian cancer, *FABP4* was identified as a key regulator of metastasis and was associated with poorer prognosis (41). *FABP4* was also previously reported to convert T4 to T3 in adipocytes, mediating adaptive thermogenesis (42). In PTC, it was found that *FABP4* is downregulated and partially mediates the tumor-suppressive effect of *PROX1* (43). In our study, *FABP4* was found to be associated with a short PFI in PTC. The tumor suppressor effect of *FABP4* in PTC and its molecular mechanisms deserve further investigation.

The roles of *PLP2, TGFBR3*, and *FXYD6* in PTC have not yet been reported. *PLP2* is a membrane protein of the endoplasmic reticulum (44). It was found to be highly expressed in glioma cells and positively associated with tumor grade and poorer prognosis. *PLP2* mediates tumor proliferation, invasion, and metastasis via the p38/ERK pathway (45). In breast cancer, *PLP2* is the direct target of the tumor suppressor MiR-422a (46). Further, its upregulation promotes the proliferation of breast cancer cells. *PLP2* also plays an oncogenic role in melanoma (47, 48). The upregulation of *PLP2* in melanoma, caused by miR-664 downregulation, enhances the proliferation and metastasis of melanoma via the PI3K/AKT pathway. In hepatocellular carcinoma, an amplitude-modulated electromagnetic field was reported to inhibit the proliferation of cancer cells via *PLP2* downregulation (49). However, its role in PTC has not yet been reported. As *PLP2* was also found to be highly expressed in PTC and associated with poor prognosis in this study, its role in PTC and the underlying molecular mechanisms deserve further attention.

*TGFBR3*, also known as betaglycan, can bind TGF-beta. It functions as a co-receptor of *TGFBR2* and also activates downstream signaling pathways in a non-canonical manner (50). Although its role in PTC has not yet been reported, it functions as a tumor suppressor in multiple cancers. For example, *TGFBR3* was found to be downregulated in prostate cancer via a loss of heterozygosity at its encoding genomic locus and epigenetic regulation (51, 52). The downregulation of *TGFBR3* also promotes the invasion and progression of prostate cancer, as well as upregulation of the prostate stem cell marker CD133 (53). In non-small cell lung cancer, *TGFBR3* is also downregulated and promotes cancer cell migration and invasion (54). In breast cancer, decreased *TGFBR3* expression was correlated with the loss of heterozygosity of its gene locus and was associated with shorter recurrence-free survival and enhanced tumor invasion, metastasis, and angiogenesis (55). In pancreatic cancer, *TGFBR3* is the target of exosomal miR-501-3p and inhibits tumor formation and metastasis (56). Similar tumor suppressor functions for *TGFBR3* have also been reported for renal cell carcinoma, endometrial carcinoma, and bladder carcinoma, among others (57, 58). In breast cancer and melanoma, loss of *TGFBR3* in dendritic cells results in altered Treg cell infiltration and the suppression of antitumor immunity, indicating its potential role in the tumor immune microenvironment (59).

*FXYD6*, located at the plasma membrane, is a member of that FXYD family, which regulates the Na+/K+-ATPase (60). *FXYD6* plays an important role in sensory organs such as the inner ear and is associated with various mental illnesses such as Alzheimer's disease (61, 62). In cancer, *FXYD6* was identified as positively associated with chemotherapy sensitivity in advanced colorectal cancer (63). *FXYD6* was also found to be upregulated in cholangiocarcinoma and hepatocellular carcinoma (64, 65). The upregulation of this marker in hepatocellular carcinoma promotes the migration and proliferation of cancer cells via the Src–ERK pathway (65). *FXYD6* was also found to be upregulated in osteosarcoma and was identified as the direct target of miR-372-3p and microRNA-137 (66). Accordingly, the upregulation of *FXYD6* reverses the tumor suppressing effects of these miRNAs in osteosarcoma (67, 68). Despite this evidence, the role of *FXYD6* in PTC and other tumors remains unknown. In our study, the downregulation of *FXYD6* was identified in PTC and was associated with worse prognosis. In addition to that in PTC, *FXYD6* was also downregulated in tumor tissues of most cancer types with a |log_2_FC| > 1 based on TCGA expression data from GEPIA (http://gepia.cancer-pku.cn), including adrenocortical carcinoma, bladder urothelial carcinoma and breast invasive carcinoma, among others. Whether *FXYD6* exerts a tumor suppressor role in these tumors and its molecular mechanisms deserves further study.

To the best of our knowledge, a prognostic model based on these five genes and the associated nomogram have not been reported to date. Our predictive model is based on the expression level of a limited number of genes, which is more economical and clinically practical than whole genome sequencing. Further, our nomogram combined with gene prognostic prediction models and clinicopathological parameters could provide clinicians with a convenient and accurate method to assess the prognosis of patients with PTC after surgery. The graphical scoring system is easy for patients to understand and is helpful to make medical decisions, thereby enabling individualized treatment.

However, our current research has some limitations. First, the main source of clinical information for our dataset was the TCGA database. The majority of patients were from North America, and thus, caution should be taken when expanding our results to patients of other ethnicities. Further, protein expression levels of the DEGs also require further investigation. Their role in the pathogenesis and progression of PTC depends on further experimental studies to elucidate the associated molecular mechanisms. The establishment and validation of the nomogram was also based on the TCGA database, and thus it is necessary to validate the clinical information and gene expression data using external datasets in future studies. Finally, the 2009 version of ATA risk stratification were used as the primary reference of evaluation in the current study since the publicly available TCGA data does not include information about the size of lymph nodes. Prospective study is required to further validate the performance of the five-gene signature and the associated nomogram using the latest version of ATA risk stratification.

## Conclusion

In our study, we established a five-gene signature and developed a prognostic nomogram in combination with prognosis-related clinical pathological parameters to predict the PFI of PTC. The five DEGs are closely related to the progression and prognosis of PTC and are thus also potential therapeutic targets. The predicted nomogram proved to be reliable in predicting the PFI of PTC and might thus be beneficial for individualized treatment and medical decision making.

## Data Availability Statement

The datasets analyzed for this study can be found in the Gene Expression Omnibus (https://www.ncbi.nlm.nih.gov/geo/) and TCGA (https://portal.gdc.cancer.gov/).

## Author Contributions

ZL and QL: conception and design. MW and HY: development of methodology. MW and XL: analysis and interpretation of data. MW: writing of the manuscript. ZL and QL: review of the manuscript. ZL: study supervision.

### Conflict of Interest

The authors declare that the research was conducted in the absence of any commercial or financial relationships that could be construed as a potential conflict of interest.
