# Supplementary material for: Identification of a Five-Gene Signature and Establishment of a Prognostic Nomogram to Predict Progression-Free Interval of Papillary Thyroid Carcinoma
Source: Front Endocrinol (Lausanne). 2019 Nov 15;10:790. doi: 10.3389/fendo.2019.00790 (PMC6872544; doi:10.3389/fendo.2019.00790)
Supplement: Supplementary file 4 [file Data_Sheet_1.pdf]

Supplementary Material

1 Supplementary Figures

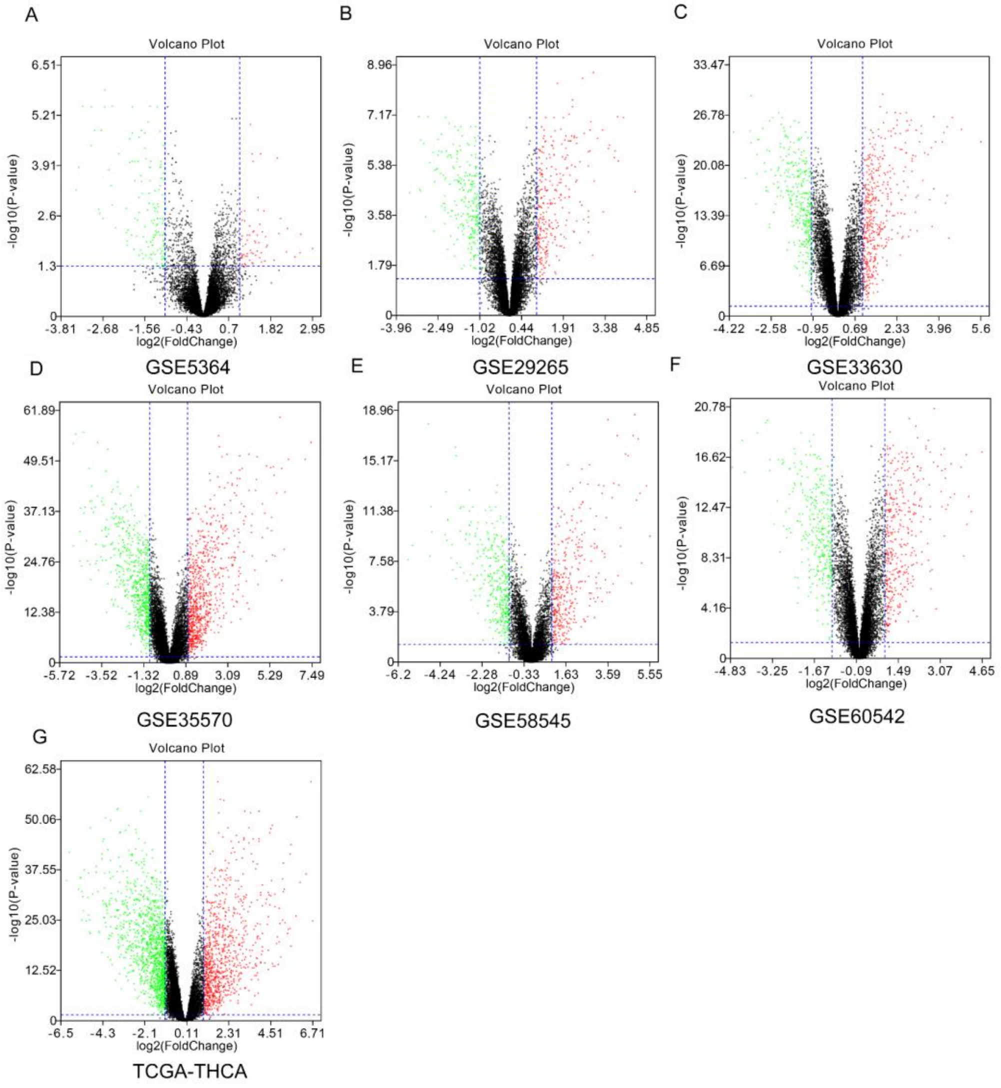

Supplementary Figure 1. Differential expression of mRNA between tumor and normal tissue in the six GEO datasets and the TCGA-THCA dataset.

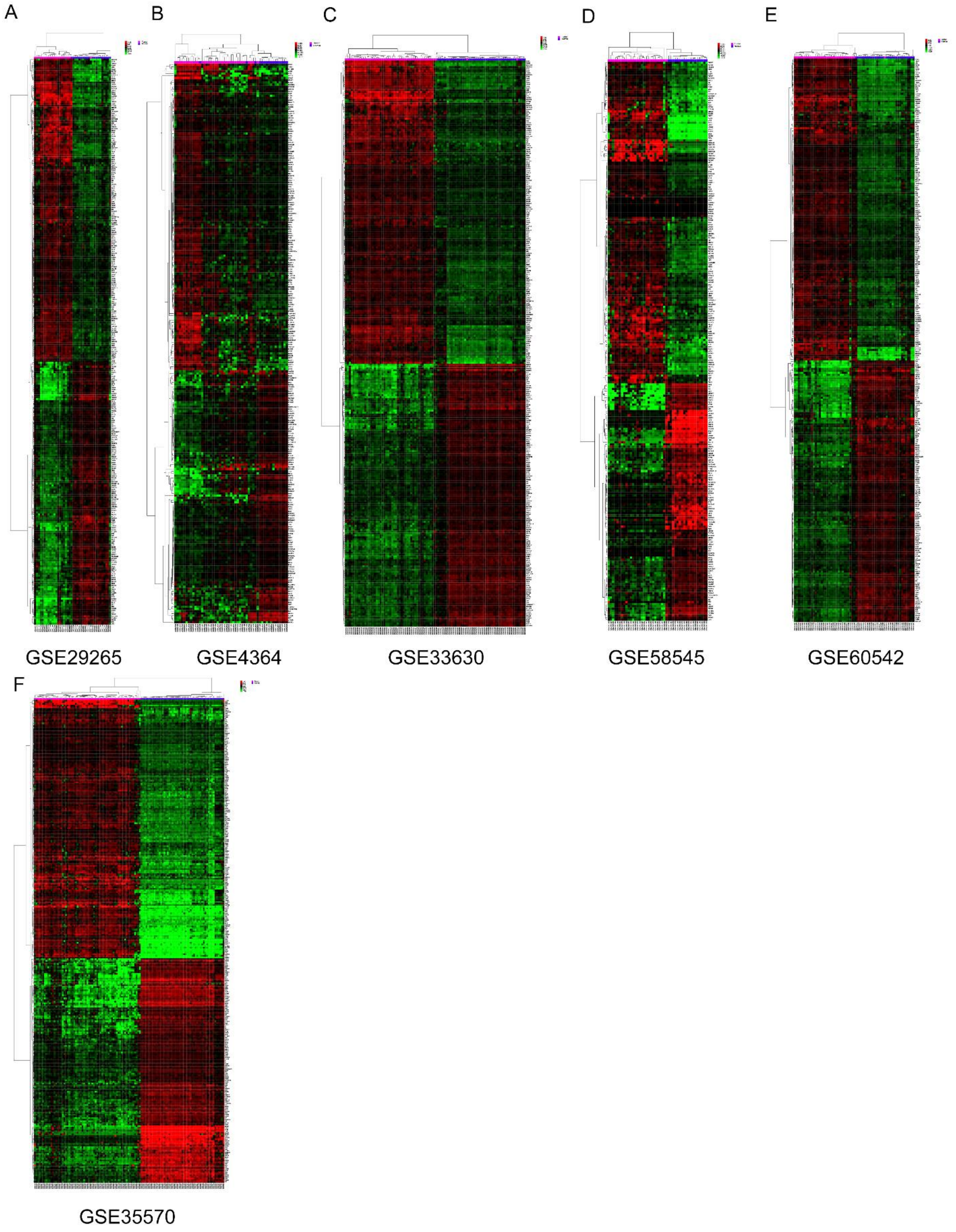

Supplementary Figure 2. Heatmaps of the 295 reliable DEGs in GSE29265, GSE4364, GSE33630, GSE58545, GSE60542 and GSE35570 dataset.

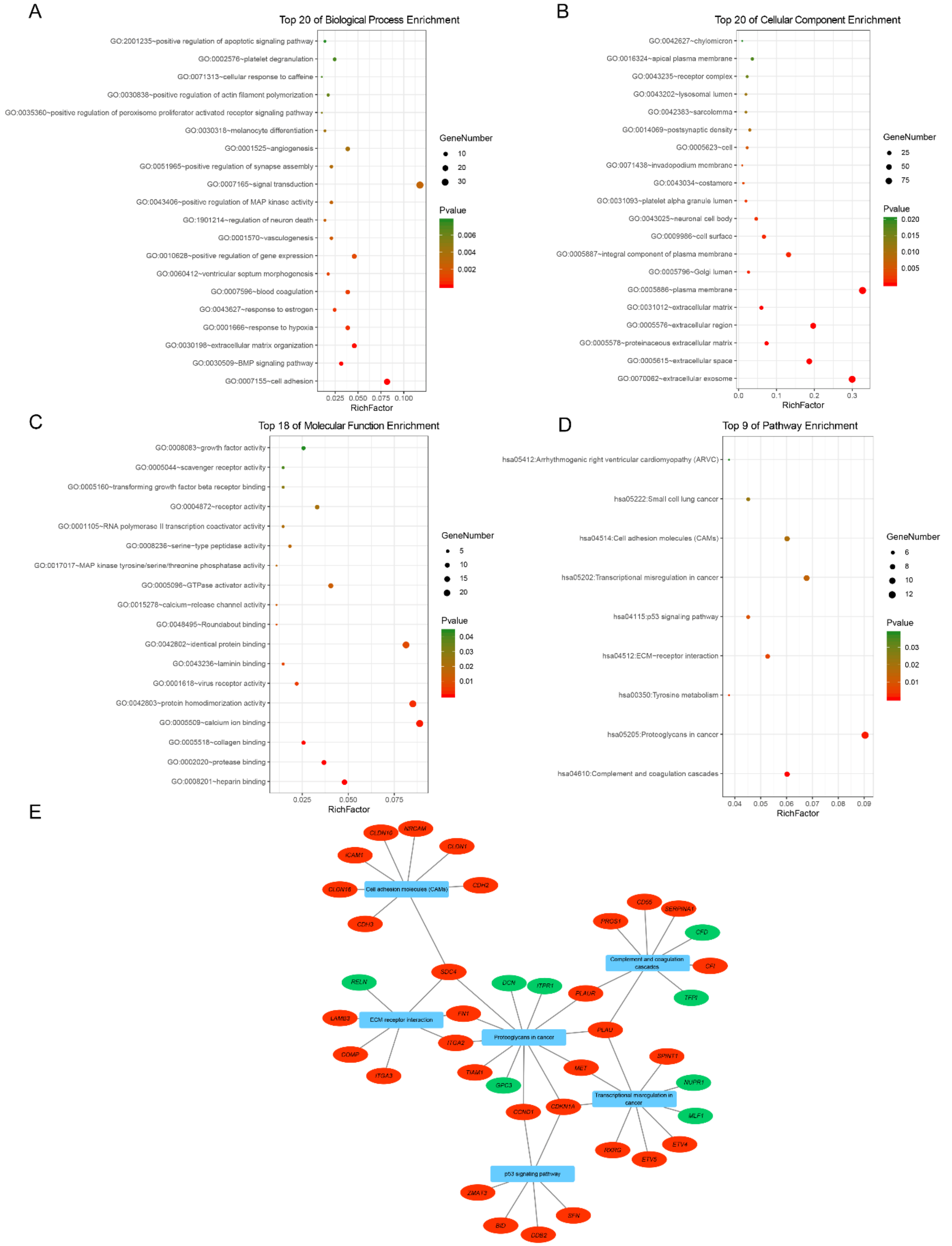

Supplementary Figure 3. Functional enrichment and pathway analysis of the differentially-expressed genes (DEGs). (A) Top 20 most enriched biological processes of the DEGs. (B) Top 20 most enriched cellular components of the DEGs. (C) Top 18 most enriched molecular functions of the DEGs. (D) Top nine most enriched pathways of the DEGs. (E)Visualization of enriched cancer related pathways and

their corresponding DEGs. Up-regulated DEGs are represented in red while down-regulated DEGs are represented in green. Pathways are represented in blue.

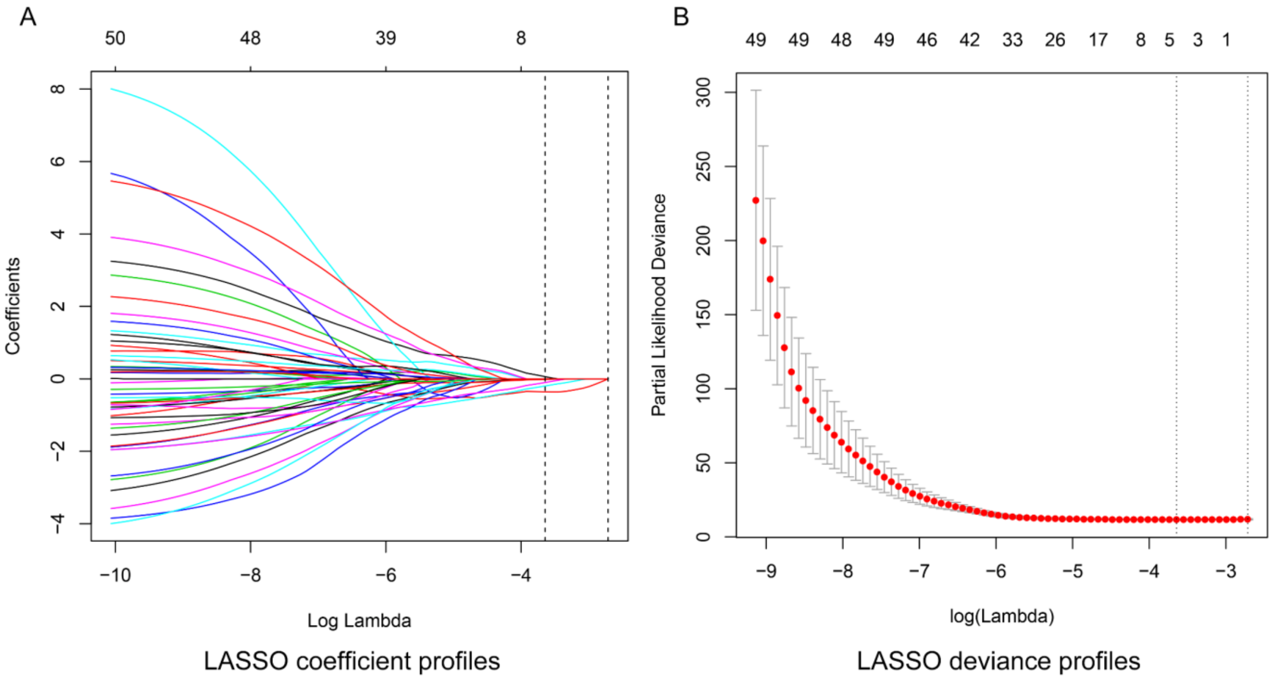

Supplementary Figure 4. LASSO analysis of the 50 prognostic DEGs in PTC. (A) LASSO coefficient profiles of the 50 prognostic DEGs in PTC. (B) Lasso deviance profiles of the 50 prognostic DEGs in

PTC. Selection criteria of Lambda was based on the value of lambda that gives minimum mean cross-validated error. Log (Lambda.min) = -3.6439.

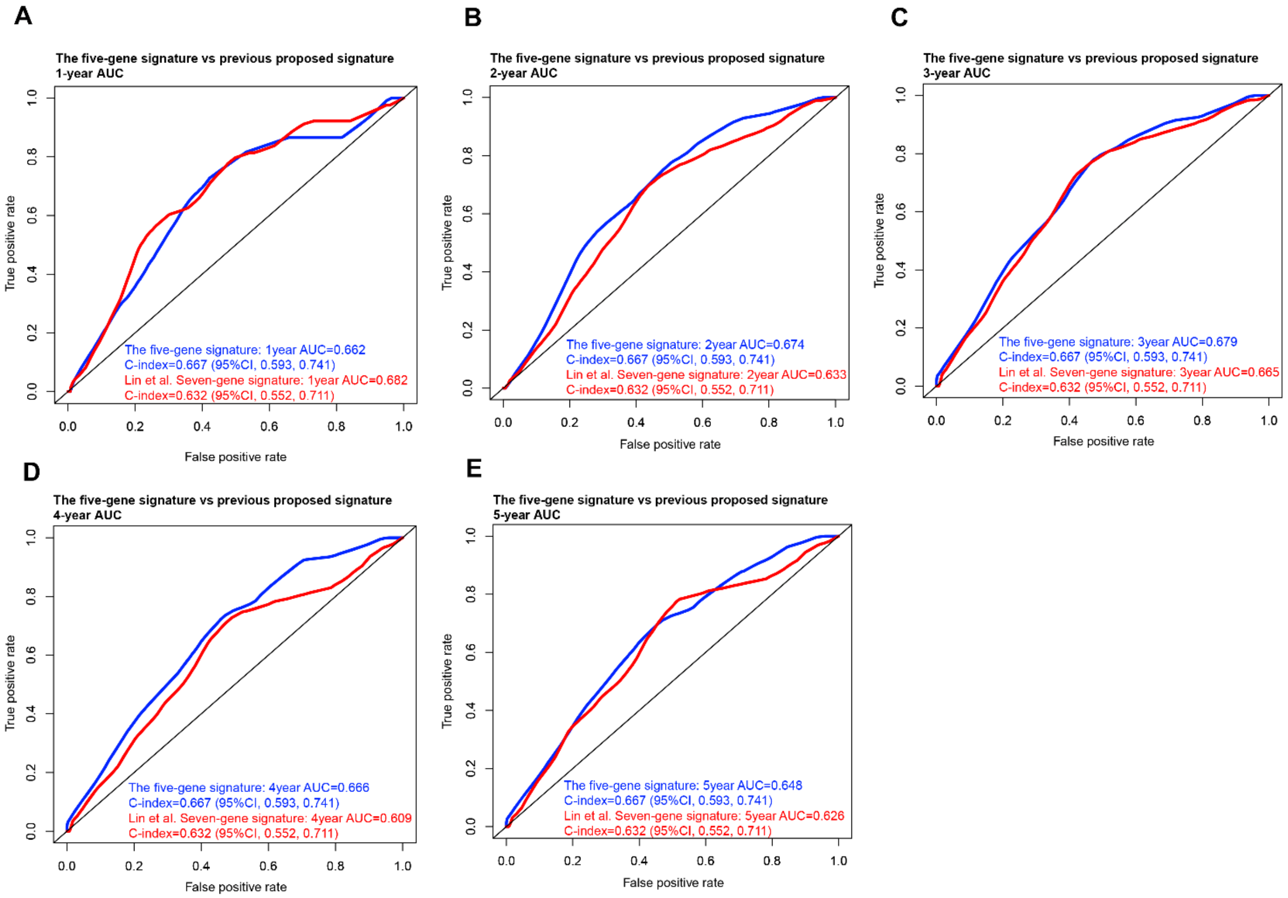

Supplementary Figure 5. (A) to (E) The prognostic performance of the five-gene signature and the seven-gene signature previously proposed by Lin et al.

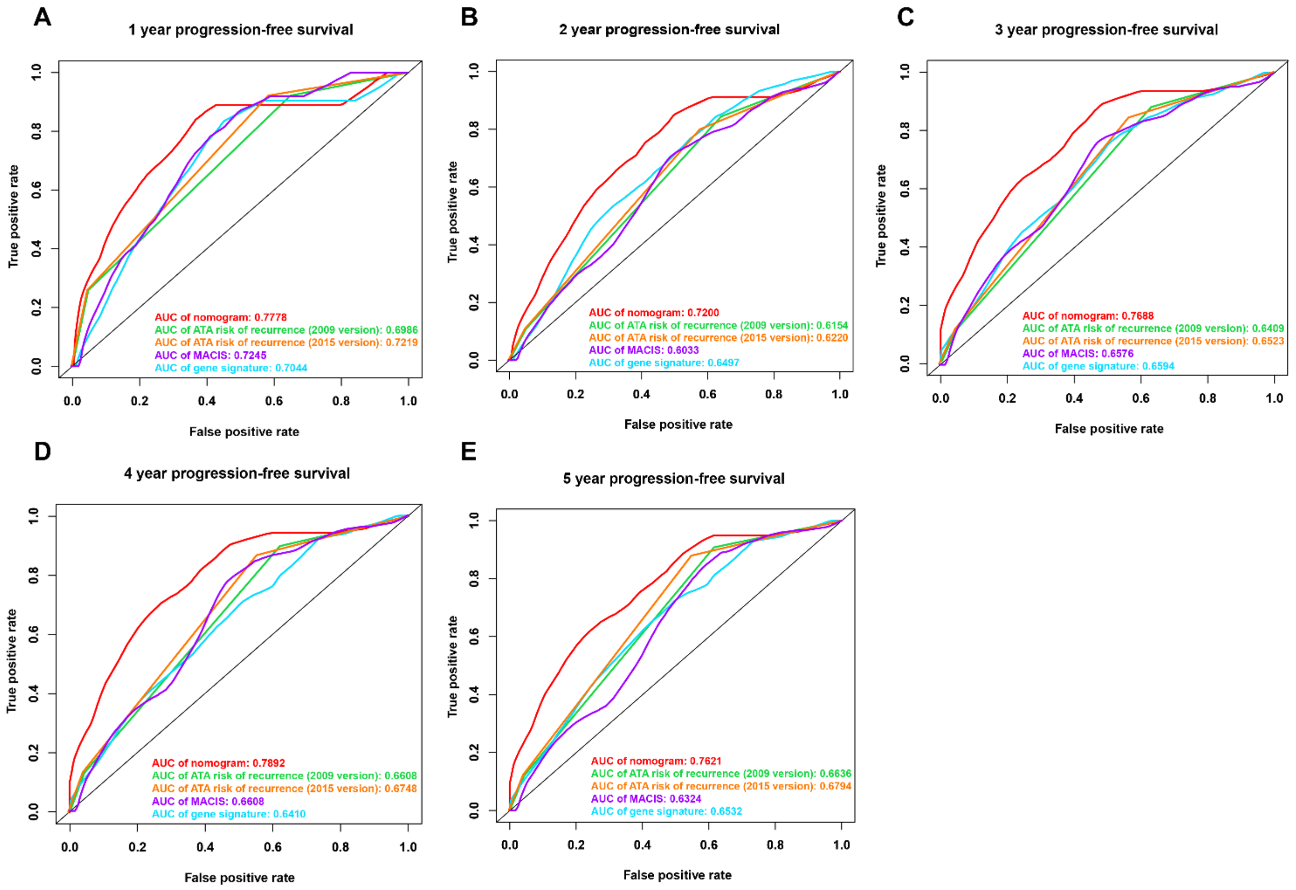

Supplementary Figure 6. (A) to (E) The prognostic performance of the five-gene signature, the gene signature-based nomogram, the 2009 version and 2015 version of American Thyroid Association (ATA) risk stratification of recurrence and the MACIS score. Modification involving lymph node size in the 2015 version of ATA risk stratification of recurrence was not included. The C-index of nomogram was 0.7747 (95%CI, 0.6870–0.8625). The C-index of 2009 version of ATA risk stratification of recurrence was 0.6377 (95%CI, 0.5601–0.7153). The C-index of 2015 version of ATA

risk stratification of recurrence was 0.6449 (95%CI, 0.5646–0.7253). The C-index of MACIS was 0.6507 (95%CI, 0.5508–0.7505).

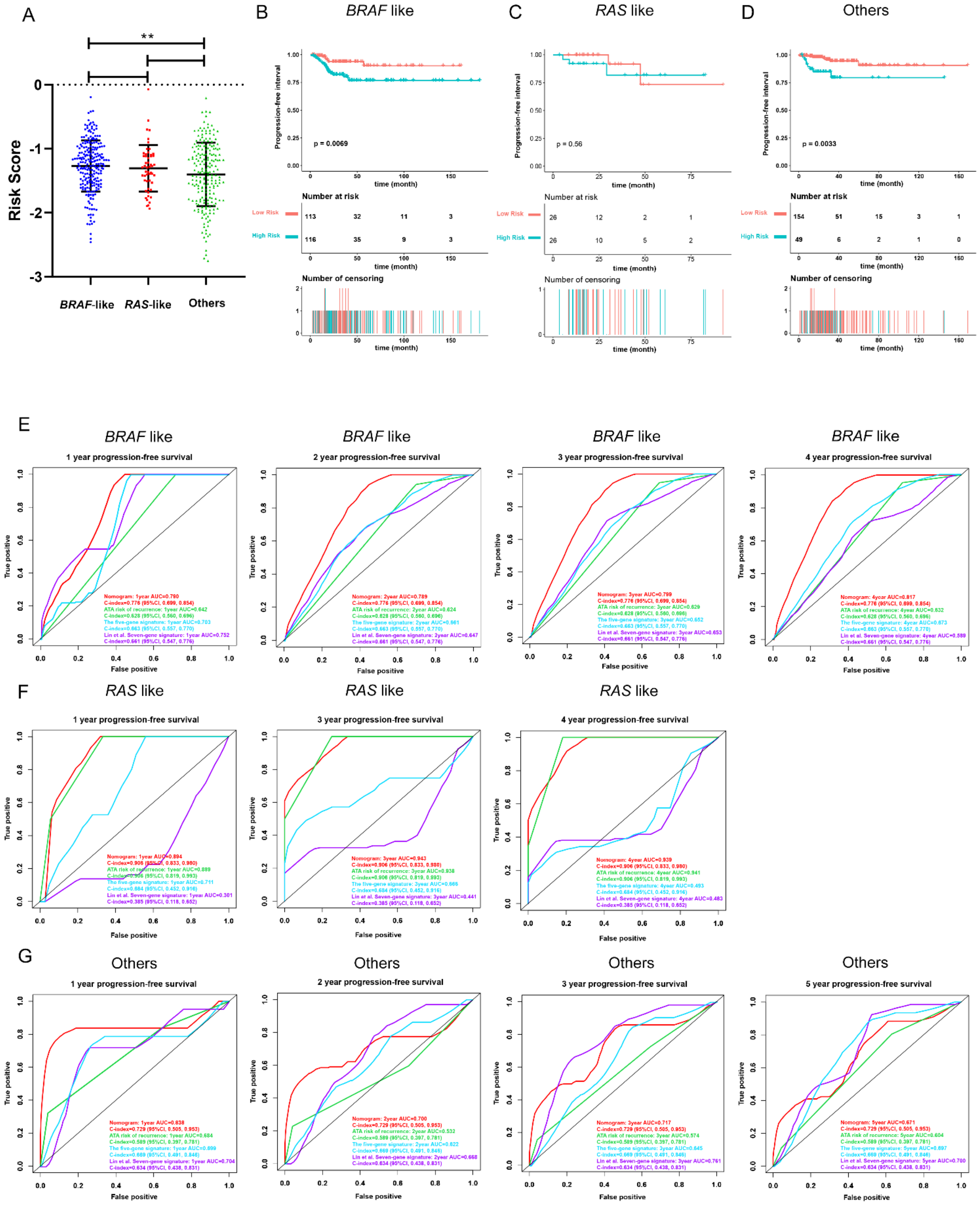

Supplementary Figure 7. (A) The distribution of the five-gene signature in different subgroups of *BRAF*-like and *RAS*-like PTC proposed by TCGA. (B) to (D) The Kaplan-Meier survival curves of the five-gene signature in different subgroups. Patients from subgroups of *BRAF*-like *RAS*-like and Others are stratified into two groups according to the optimal cutoff values for the risk scores calculated by X-Tile software. (E) to (G) The prognostic performance of the five-gene signature, the gene signature-based nomogram, the American Thyroid Association (ATA) risk stratification of recurrence and the seven-gene signature previously proposed by Lin et al in different subgroups of *BRAF*-like PTC, *RAS*-like PTC and Others.

Supplementary Tables

Supplementary table1. 295 reliable DEGs identified based on the intersection between GEO and TCGA results.

Supplementary table2. Functional enrichment analysis of the DEGs.

Supplementary table3. Reasons for exclusion from the evaluation of prognostic factors and establishment of nomogram.
